# Effect of residual extractable lignin on acetone–butanol–ethanol production in SHF and SSF processes

**DOI:** 10.1186/s13068-020-01710-2

**Published:** 2020-04-10

**Authors:** Jing Li, Yu Zhang, Suan Shi, Maobing Tu

**Affiliations:** 1 Alabama Center for Paper & Bioresource Engineering, Auburn University, Auburn, AL 36849 USA; 2 Engineering Laboratory for AgroBiomass Recycling & Valorizing, College of Engineering, China Agricultural University, Beijing, 100083 China; 3 Department of Chemical and Environmental Engineering, University of Cincinnati, Cincinnati, OH 45221 USA

**Keywords:** Fermentation, Residual extractable lignin, Acetone–butanol–ethanol, Acid crash

## Abstract

**Background:**

Lignin plays an important role in biochemical conversion of biomass to biofuels. A significant amount of lignin is precipitated on the surface of pretreated substrates after organosolv pretreatment. The effect of this residual lignin on enzymatic hydrolysis has been well understood, however, their effect on subsequent ABE fermentation is still unknown.

**Results:**

To determine the effect of residual extractable lignin on acetone–butanol–ethanol (ABE) fermentation in separate hydrolysis and fermentation (SHF) and simultaneous saccharification and fermentation (SSF) processes, we compared ABE production from ethanol-washed and unwashed substrates. The ethanol organosolv pretreated loblolly pine (OPLP) was used as the substrate. It was observed that butanol production from OPLP-UW (unwashed) and OPLP-W (washed) reached 8.16 and 1.69 g/L, respectively, in SHF. The results showed that ABE production in SHF from OPLP-UW prevents an “acid crash” as compared the OPLP-W. In SSF process, the “acid crash” occurred for both OPLP-W and OPLP-UW. The inhibitory extractable lignin intensified the “acid crash” for OPLP-UW and resulted in less ABE production than OPLP-W. The addition of detoxified prehydrolysates in SSF processes shortened the fermentation time and could potentially prevent the “acid crash”.

**Conclusions:**

The results suggested that the residual extractable lignin in high sugar concentration could help ABE production by lowering the metabolic rate and preventing “acid crash” in SHF processes. However, it became unfavorable in SSF due to its inhibition of both enzymatic hydrolysis and ABE fermentation with low initial sugar concentration. It is essential to remove extractable lignin of substrates for ABE production in SSF processes. Also, a higher initial sugar concentration is needed to prevent the “acid crash” in SSF processes.

## Background

Lignocellulosic biomass has great potential to replace petroleum-based liquid fuels and chemicals, thereby addressing our national needs for energy independence and domestic jobs, as well as environmental issues [1–3]. Butanol is one of the promising alternative biofuels, which can be produced from biomass [4]. Butanol production along with acetone and ethanol from sugars by *Clostridium* is known as “acetone–butanol–ethanol (ABE) fermentation” [5]. The fermentation produces butyrate and acetate at the beginning (acidogenic phase), in which the excess electrons are used to reduce H^+^ to H_2_. Butanol, acetone, and ethanol start to be produced in the second phase (solventogenic phase) [6, 7]. Solventogenesis typically is accompanied by sporulation. In batch ABE fermentation, “acid crash” occasionally occurred when fermentation is performed without pH control [8]. When this occurs, excess of acids is produced and the switch from the acidogenic phase to the solventogenic phase stopped [9]. The glucose consumption, acid production, and ABE generation are also terminated [8]. Previous studies suggested “acid crash” takes place with *C. beijerinckii* under the high concentration of undissociated acids (57–60 mM) [8]. It has been reported that solvent production by *C. acetobutylicum* ceased when formic acid accumulated to 0.5–1.24 mM [10]. To prevent the “acid crash”, several strategies have been developed by introducing pH control or by lowering the metabolic rate [11, 12]. Buffering the initial pH at 5.0 (with sodium acetate) produced the highest butanol concentration of 12.3 g/L by *C. acetobutylicum* at 72 h [12]. Incubation *C. carboxidivorans* at a lower temperature of 25 °C resulted in higher alcohol titers due to the lower metabolic rates [11]. Lowering yeast extract concentration (nutrients) reduced acid production rate and enabled solventogenesis to persist for a longer time with higher ABE concentration [8]. As a result, “acid crash” can be prevented by lowering the acid production rate or by providing less desirable growing conditions.

As far as we know, “acid crash” in ABE fermentation with lignocellulosic biomass has not been reported. ABE fermentation from enzymatic hydrolysate of pretreated corncobs has shown higher ABE yield and butanol concentration (12.3 g/L) than mixed sugar control, which indicated hydrolysates may contain stimulating compounds to improve ABE fermentation [13]. Different processes have been used to ferment pretreated biomass to butanol, including separate hydrolysis and fermentation (SHF) and simultaneous saccharification and fermentation (SSF). Sasaki et al. reported an ABE production of 15.29 g/L in SHF process versus 13.41 g/L in SSF process from steam-exploded wood chips [14]. Butanol production from wheat straw by SSF using *C. beijerinckii* has been reported to produce 21.4 g/L ABE [15], and it should be noted pretreated substrates have been washed with water. The effect of lignin presence on butanol production by *C. acetobutylicum* has been investigated with cellobiose as the carbon source, in which they found lignin (1 g/L) delayed and decreased butanol production and promoted the accumulation of acetic and butyric acids [16].

Due to the low temperature limiting the hydrolysis rate in SSF, the productivity might be lower than the fermentation step in SHF. It is reported butanol production in SSF was 24 h slower than SHF since the sugar in SHF process is readily available to initiate the acid production once the clostridia inoculum was induced [17]. It has been reported the reassimilation of acids to solvents ceased when the remaining sugar was low and thus more acids were observed when lower sugar was applied [17, 18]. The low sugar concentration might also affect the phase transition from acidogenesis to solventogenesis in SSF process [17]. So, to increase the available sugar in SSF is critical to ABE fermentation, which may be achieved by increasing the substrate concentration and enzyme activity [19], improving the pretreatment efficiency to increase the accessibility of substrate, and supplementing sugar-rich prehydrolysates.

The objective of this study is to assess the effect of residual lignin in organosolv-pretreated substrates on ABE production. The effect of lignin on enzymatic hydrolysis has been extensively studied due to its strong interaction with enzymes [20, 21]. Previous studies reported that ethanol organosolv lignin (EOL) should be maintained in substrates and solvent washing after pretreatment was not necessary for enzymatic hydrolysis [22]. However, the impact of residual lignin on subsequent microbial fermentation was not well understood. Recently, Li et al. reported a negative correlation between lignin level and ethanol production, indicating the inhibitory effect of lignin on ethanol fermentation [23]. In this study, the effect of ethanol washing on ABE production from organosolv-pretreated loblolly with SHF and SSF processes will be examined. It is hypothesized that the residual extractable lignin (similar to EOL) on pretreated substrates can prevent the “acid crash” in ABE fermentation by lowering the metabolic rate and reducing the acid production rate. It is also possible that the lower temperature (35 °C) in SSF process can slow down the enzymatic hydrolysis and prevent the “acid crash”. The effect of ethanol washing on enzymatic hydrolysis of organosolv-pretreated loblolly pine (OPLP) will be compared between the washed substrates (OPLP-W) and the unwashed substrates (OPLP-UW). The effect of ethanol washing on ABE production in SHF and SSF processes will be also compared between OPLP-W and OLPL-UW. In addition, the detoxified prehydrolysates will be supplemented into SSF process to evaluate its effect on ABE fermentation.

## Results and discussion

### Effect of ethanol washing on enzymatic hydrolysis of OPLP

**Fig. 1:**
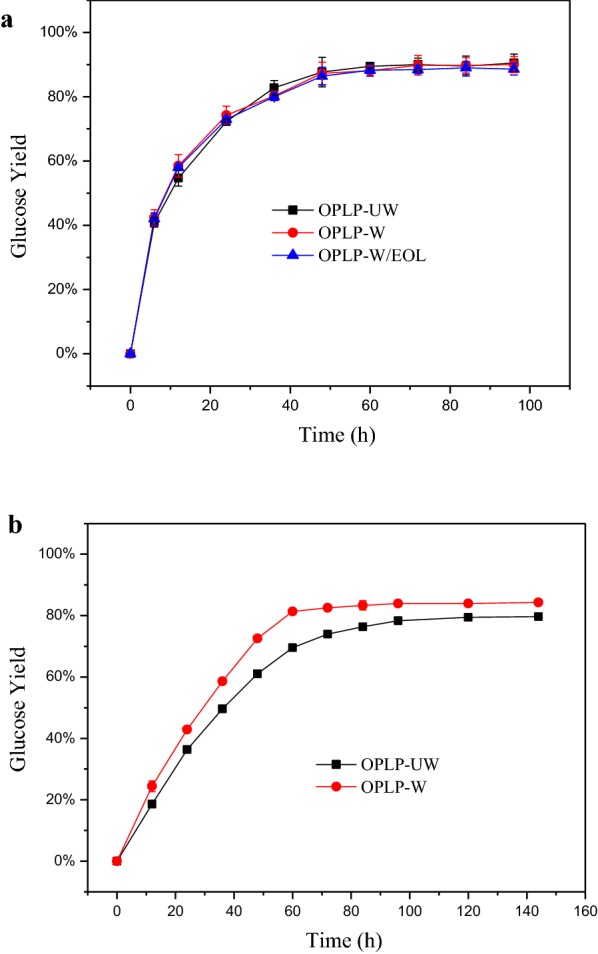
Effect of ethanol washing on enzymatic hydrolysis of OPLP: **a** 50 °C and pH 4.8 and **b** 35 °C and pH 6.0

The ethanol extractives content (9.64%) in OPLP-UW were much higher than those in the untreated biomass (1.18%) (Table 2). The ethanol extractives were reduced to 0.79% in OPLP after ethanol washing (Table 2). During the organosolv pretreatment, lignin was depolymerized and a significant amount of β-o-4 linkages was cleaved which was catalyzed by acids [24, 25]. The depolymerized lignin was precipitated on the surface of the wood fibers and it can be largely removed by ethanol washing [22, 26]. Lai et al. have reported that the ethanol-washed extractives were similar to ethanol organosolv lignin (EOL) by ^13^C-NMR [22]. To examine the effect of ethanol washing on enzymatic digestibility of OPLP, the pretreated substrates with and without ethanol washing were hydrolyzed and compared under the SHF and SSF conditions (Fig. 1). The results showed the 72-h hydrolysis yield of OPLP-W and OPLP-UW was similar (90%) (Fig. 1a). The addition of precipitated organosolv lignin (0.3 g) also did not change the hydrolysis yield of OPLP-W. It indicated that ethanol washing did not have any positive or negative effects on substrates digestibility at the SHF conditions (50 °C and pH 4.8). Previous studies reported that EOL from loblolly pine had a negative effect on enzymatic hydrolysis of OPLP, in which the enzyme loading was 5 FPU/g glucan [27]. High enzyme loading (25 FPU/g glucan) in this study probably overcame the negative effect of EOL on enzymatic hydrolysis. It reduced the effect of nonproductive binding between cellulase and lignin by providing sufficient enzyme active sites [28]. The residual lignin adsorbed on the additional active sites offered by extra enzyme compared to low enzyme loading and resulted in the negligible negative effect of residual lignin on enzymatic hydrolysis. Enzyme dosage below 10 FPU/g cellulose is usually considered as low enzyme loading [29, 30]. The National Renewable Energy Laboratory (NREL) in the United States set the enzyme loading to 19–33 mg protein/g cellulose (equivalent to 15–20 FPU/g cellulose) when building the ethanol cost evaluation model, which is the normal range for bioconversion of lignocellulosic biomass [31, 32]. A minimum cellulase (Celluclast 1.5) loading of 32 mg protein/g cellulose is required for efficient hydrolysis (70% glucan conversion) of organosolv-pretreated lodgepole pine [33]. A slightly high enzyme loading (25 FPU/g glucan) applied in this study is to minimize the rate-limiting effect of enzymatic hydrolysis during the SSF process. Therefore, by eliminating the enzyme hydrolysis impact on SSF, the effect of extractable lignin on ABE production could be explored. However, the practice of reducing enzyme loading could be carried out in the future upon obtaining a better understanding of how the extractable lignin affects ABE production in SHF and SSF processes.

While for the enzymatic hydrolysis of OPLP-W and OPLP-UW at the SSF conditions (35 °C and pH 6), the 72 h hydrolysis yield of OPLP-W and OPLP-UW was 82.5% and 73.9%, respectively (Fig. 1b), which were lower than those at the SHF conditions (50 °C and pH 4.8). Notably, the OPLP-UW had even lower hydrolysis yield compared to OPLP-W under the test conditions. The lower temperature (35 °C) also resulted in the lower initial hydrolysate rate (Fig. 1). According to the adsorption kinetics of cellulase on the cellulose and lignin, the decrease of temperature reduced the adsorption of cellulase on both cellulose and lignin, however, the reduction was much more considerable for cellulose than lignin [28, 34]. The lignin had a higher affinity to cellulase than cellulose [28, 35]. These factors made more cellulase adsorbed on lignin rather than cellulose and the negative effect of extractable lignin on enzymatic hydrolysis was exhibited at the lower temperature. In addition to the lower temperature, higher pH (6.0) could be another main reason for lower hydrolysis yield and initial rate. Similar results have been reported that higher pH (6.0) reduced the hydrolysis yield of organosolv-pretreated loblolly pine at 10 FPU/g glucan [36]. The pH increase from 5 to 7 could result in less adsorption of cellulase on cellulose substrate [34] and decrease the enzyme activity; pH 4.8 has long been suggested for cellulase enzymatic hydrolysis [37]. Adding CaCO_3_ to control pH in the hydrolysis solution could also contribute to the decrease in enzyme activity. It is reported that the inhibition of CaCO_3_ to enzymatic hydrolysis possibly caused by nonproductive enzyme binding on CaCO_3_ particles and deactivation of enzyme resulting from enzyme aggregation by dissociated calcium ion [38, 39]. The observation that the removal of extractable lignin from the examined substrate (softwood) by ethanol washing improved the enzymatic hydrolysis appears not to agree with the effect of EOL from hardwood, but is consistent with the effect of EOL from softwood by Lai et al. [22, 40]. They reported a contrasting effect of hardwood and softwood organosolv lignin, where EOL from hardwood enhanced enzyme hydrolysis and EOL from softwood inhibited enzymatic hydrolysis. Huang et al. investigated the reason why the lignin from two types of sources exerted opposite effects [27]. They found a strong correlation between hydrophobicity and zeta potential of EOL and enzymatic hydrolysis yield, indicating the stimulation or inhibition effect of lignin is controlled by the combination of hydrophobicity and zeta potential.

### Effect of ethanol washing on ABE production in SHF processes

**Fig. 2:**
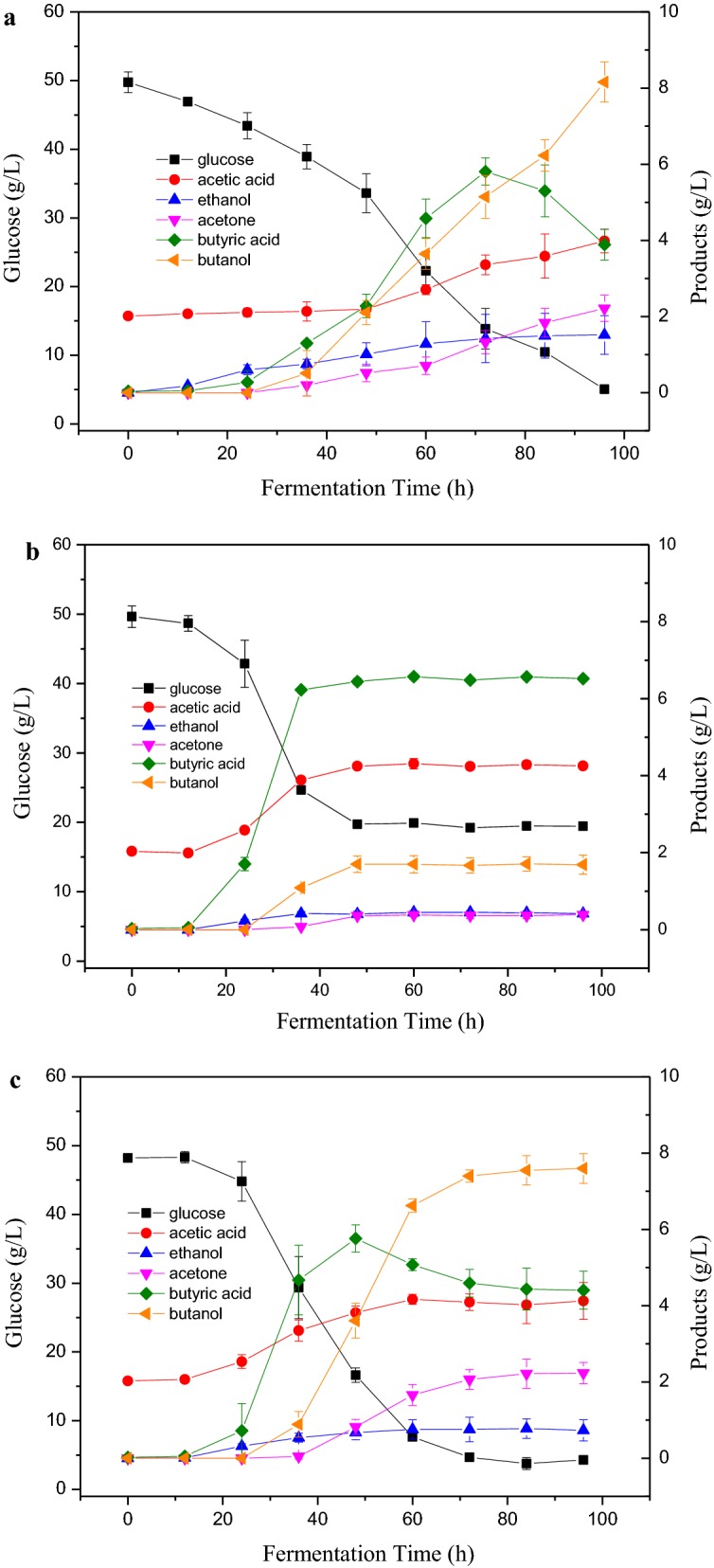
ABE fermentation of the hydrolysates from OPLP-UW (**a**), OPLP-W (**b**) and OPLP-W/EOL (**c**)

Under SHF conditions, the ethanol washing showed no effect on the 72-h hydrolysis yield of organosolv-pretreated loblolly pine at current enzyme loading (25 FPU/g glucan). However, the subsequent effect on ABE fermentation of the hydrolysates from ethanol-washed substrates is unknown. Therefore, three enzymatic hydrolysates from OPLP-UW, OPLP-W and OPLP-W/EOL (plus precipitated EOL) were compared in ABE fermentation (Fig. 2). It was observed that butanol production from the OPLP-UW hydrolysate was 8.16 g/L with a yield of 0.14 g/g at 96 h, and the residual glucose was 5.06 g/L (Fig. 2a). The initial glucose consumption rate (within 36 h) was low at 0.30 g/L/h. The organism began to accumulate butyric acid at 24 h and acetic acid at 48 h. Butyric acid peaked (5.81 g/L) at 72 h and then gradually decreased to 3.89 g/L at 96 h. Butanol production began late at 36 h in the fermentation. The acetone and ethanol reached 2.22 and 1.52 g/L at 96 h, respectively. While for the ABE fermentation with the OPLP-W (Fig. 2b), the initial glucose consumption rate (within 36 h) was fast at 0.69 g/L/h, but glucose consumption and ABE production ceased at 48 h. The initial glucose concentration was nearly 50 g/L. The organism began to accumulate butyric acid at 12 h and quickly reached 6.23 g/L at 36 h and did not decrease further. Butanol production began from 36 h, but stopped at 48 h with a low concentration of 1.69 g/L. The butyric acid and acetic acid were 6.44 g/L and 4.24 g/L at 48 h and then leveled off. The residual glucose was 19.72 g/L at 48 h and did not change further. It indicated ABE production from the OPLP-W hydrolysate suffered an “acid crash”, in which solventogenesis was initiated but the metabolic activity (glucose consumption, acid production, and ABE production) ceased within a short time (Fig. 2b). The butyric acid production rate (2.92 mM/h, between 12 and 36 h) was much higher than that (0.59 mM/h) in ABE fermentation with OPLP-UW hydrolysate. The toxic butyric acid was generated quickly inside cells and inhibited solventogenesis and ceased the ABE production. It has been suggested “acid crash” occurs in pH-uncontrolled ABE fermentation when undissociated acids exceed 57–60 mM [8]. In this study, pH was controlled by CaCO_3_ and the pH was kept in the range of 5 to 6 over the fermentation time. The concentration of the total acids reached 144 mM at 48 h, which included undissociated acids and dissociated acids. It has been proposed previously that the high concentration of dissociated acids rather than undissociated acids are responsible for the inhibition of solventogenesis at some ABE fermentation [8]. The comparison of ABE fermentation with OPLP-W and OPLP-UW hydrolysates indicated that the metabolism of the organism could be altered by ethanol washing or the presence of extractable lignin (after pretreatment). We hypothesized that extractable lignin (similar to EOL) can inhibit the glucose consumption and acid production rates thus prevent the “acid crash” in ABE fermentation. To test this hypothesis, precipitated EOL from organosolv pretreatment was added into ABE fermentation of the OPLP-W hydrolysates (Fig. 2c). The initial glucose consumption rate (within 36 h) was fast at 0.52 g/L/h, but glucose consumption and ABE production continued until 84 h. The organisms began to produce butyric acid at 12 h and increased to 4.67 g/L at 36 h and reached 5.76 g/L at 48 h, then decreased due to the shift from acidogenic phase to solventogenic phase. Butanol production began from 36 h and reached 7.60 g/L at 96 h. The acetic acid was 4.17 g/L at 60 h and then leveled off. The acetone and ethanol reached 2.23 and 0.73 g/L at 96 h, respectively. The residual glucose was 4.31 g/L at 96 h, which was similar to that from the ABE fermentation of OPLP-UW hydrolysates. The results demonstrated that the presence of extractable lignin could lower the metabolic rate and prevent the “acid crash” in ABE fermentation. Different approaches have been suggested previously to prevent “acid crash” by pH controlling or lowering the metabolic rate. Lowering yeast extract concentrations (0.05 g/L) in the medium resulted in higher ABE production of 134 mM, low sugar uptake and acid product rates [8]. Overexpressing aldehyde/alcohol dehydrogenase and CoA-transferase in *Clostridium beijerinckii* was able to prevent “acid crash” and increase butanol production [41]. Syngas fermentation with *Clostridium carboxidivorans* at a low temperature has been reported to enhance butanol production by lowering metabolic rates at 25 °C [11]. In this study, we found the inhibitory extractable lignin could be potentially effective to prevent the “acid crash” in ABE fermentation by lowering the glucose uptake and acid production rates.

### Effect of ethanol washing on ABE production in SSF processes

**Fig. 3:**
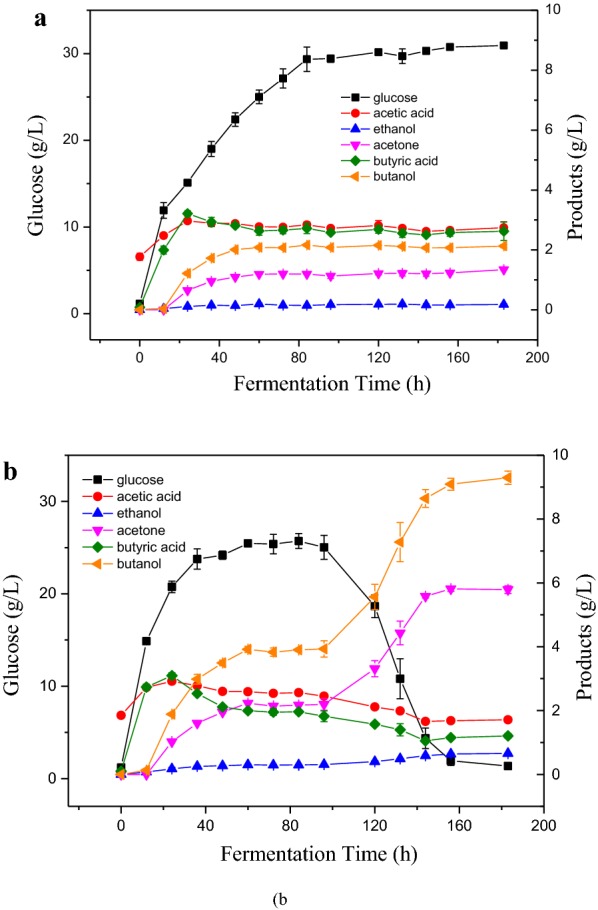
ABE production with OPLP-UW (**a**) and OPL P-W (**b**) in SSF process

ABE production with OPLP-UW and OPLP-W in SSF was compared (Fig. 3 and Table 1). In both cases, ABE fermentation suffered “acid crash” after 60 h, and butanol, ethanol, and acetone production ceased. However, the ABE production recommenced at 96 h for OPLP-W. Specifically for OPLP-UW, acetic acid and butyric acid quickly reached 2.97 and 3.21 g/L at 24 h, respectively. The butanol reached 1.22 g/L at 24 h. The glucose concentration reached 15.11 g/L at 24 h and it was much lower than the initial glucose concentration in the SHF process. For OPLP-W, acetic acid and butyric acid reached 2.92 and 3.09 g/L at 24 h, respectively, which are similar to those in OPLP-UW. The butanol reached 1.89 g/L at 24 h. The glucose concentration (20.75 g/L) was 37% higher than that in OPLP-UW at 24 h. This suggested that ethanol washing significantly increased the hydrolyzability of OPLP-W as compared to OPLP-UW, which provided more initial glucose in SSF process. Cells produced more butanol (3.92 g/L) and less butyric acid (2.00 g/L) from OPLP-W than that from OPLP-UW (2.08 g/L butanol and 2.63 g/L butyric acid) at 60 h. This indicated that initial sugar concentration significantly affected the solvent and acid production; cells appear to produce more acids and fewer solvents when the initial sugar concentration is low. A similar observation has been reported previously, where only 2.93 g/L of solvents were produced from 20 g/L of glucose as compared to 8.77 g/L of solvents from 40 g/L of glucose [42].

**Table 1:** Acetone–butanol–ethanol fermentation in SHFand SSF

	SHF			SSF		
	OPLP-UW	OPLP-W	OPLP-W/EOL	OPLP-UW	OPLP-W	OPLP-W/PH^a^
Residual glucose (g/L)	5.06 ± 0.13	19.42 ± 0.51	4.30 ± 0.25	30.93 ± 0.02	1.36 ± 0.33	0.59 ± 0.36
Butanol (g/L)	8.16 ± 0.53	1.69 ± 0.25	7.60 ± 0.39	2.13 ± 0.05	9.29 ± 0.21	10.51 ± 0.18
Butanol yield (g/g)	0.14 ± 0.01	0.03 ± 0.00	0.13 ± 0.01	0.04 ± 0.00	0.16 ± 0.00	0.15 ± 0.00
ABE (g/L)	11.89 ± 0.12	2.66 ± 0.33	10.56 ± 0.22	3.65 ± 0.05	15.74 ± 0.33	18.29 ± 0.22
ABE yield (g/g)	0.20 ± 0.00	0.04 ± 0.01	0.18 ± 0.00	0.06 ± 0.00	0.27 ± 0.01	0.26 ± 0.01
Butyric acid (g/L)	3.89 ± 0.41	6.52 ± 0.07	4.41 ± 0.50	2.62 ± 0.31	1.21 ± 0.07	1.68 ± 0.04
Acetic acid (g/L)	3.99 ± 0.31	4.25 ± 0.05	4.13 ± 0.48	2.74 ± 0.20	1.71 ± 0.06	1.80 ± 0.01
Acid crash	No	Yes	No	Yes	Yes^b^	No

Data are presented as the final point in fermentation processes, SHF, 96 h; SSF for OPLP-UW and OPLP-W, 183 h; SSF for OPLP-W/PH, 132 h. The value was presented as mean value ± standard deviation

^a^OPLP-W/PH: ethanol-washed OPLP with detoxified prehydrolysates

^b^Fermentation recommenced after acid crash

In addition, the metabolic activity including acid production and ABE production ceased at 60 h for OPLP-UW, the glucose uptake probably also ceased (Fig. 3a). The total acid concentration was 76 mM at 60 h and did not change until 183 h. The residual extractable lignin not only inhibited the enzymatic hydrolysis, but also inhibited the microbial fermentation. Unexpectedly for OPLP-W, the solventogenesis and glucose uptake recommenced were at 96 h. All the glucose was consumed, and the final butanol concentration reached 9.29 g/L at 183 h. The total ABE concentration reached 15.74 g/L. During the phase of metabolic inactivity (60–96 h), the total acid concentration had slowly decreased from 66 to 61 mM [8]. Similarly, ABE recommencement after “acid crash” has been reported on pure glucose fermentation before, when the total undissociated acids dropped below a threshold of 55 mM. It should be noticed that the final acetone concentration reached 5.79 g/L, which was much higher than those in the SHF process. The results indicated that residual extractable lignin in OPLP-UW inhibited ABE fermentation and potentially intensified “acid crash” in the SSF processes. Comparing the ABE fermentation in SHF and SSF processes, the effect of residual extractable lignin was beneficial in SHF on ABE production by slowing the glucose consumption in ABE fermentation at high initial glucose concentration (50 g/L), but it became unfavorable in SSF due to its inhibition on both enzymatic hydrolysis and ABE fermentation with low initial sugar concentration (around 0 g/L). In SHF processes, high sugar concentration (50 g/L) was available for fast acidogenesis. The presence of extractable lignin in OPLP-UW hydrolysate inhibited microbial metabolic activity and decreased the metabolic rate. Subsequently, the “acid crash” was avoided in OPLU-UW and OPLP-W/EOL hydrolysates. In this case, extractable residual lignin helped ABE fermentation in SHF processes. In SSF processes, the low initial sugar concentration resulted in an “acid crash” for both OPLP-UW and OPLP-W substrates after 60 h. The inhibition of extractable lignin on enzymatic hydrolysis of OPLP-UW made it even less favorable for ABE production due to the lower sugar concentration. The inhibition of extractable lignin on microbial metabolic activity further intensified the “acid crash” for OPLP-UW. This suggested that inhibitory extractable lignin could deep “acid crash” in low sugar concentration for ABE production. Without the presence of extractable lignin in OPLP-W, the butyric and acetic acids were slowly consumed in the “acid crash” phase, which in turn enabled the solventogenesis and glucose uptake to recommence at 96 h. Therefore, it is essential to remove extractable lignin of substrates for ABE production in SSF processes. And a higher initial sugar concentration is needed to prevent the “acid crash” in the SSF processes.

The residual lignin was observed to aid ABE production in the SHF process, but hinder the ABE production in the SSF process. It is considered to affect the occurrence of “acid crash” together with initial sugar concentration. However, the threshold of sugar concentration that resulted in “acid crash” in both SHF and SSF is not clear and of interest. In the meantime, the presence of lignin levels is also a critical variable affecting the onset of “acid crash”. The combination effect of lignin and initial sugar concentration was also examined in the next part of experiment with the addition of prehydrolysates. Under the test experiment, it is estimated the initial sugar concentration between 5 and 20 g/L could potentially avoid the acid crash in SHF or SSF processes. The initial sugar concentration in SHF or SSF could be changed by varying solid loading. It is speculated that the ABE production from OPLP-W might be higher than OPLP-UW in SHF process when the solid loading is lower than the current study. Also, the “acid crash” might be avoided by increasing the solid loading of OPLP-W and improving the enzymatic hydrolysis in SSF process. The improvement of enzymatic hydrolysis could be achieved by increasing enzyme dosage or adding additives.

### Effect of adding detoxified prehydrolysates on ABE fermentation in SSF processes

**Fig. 4:**
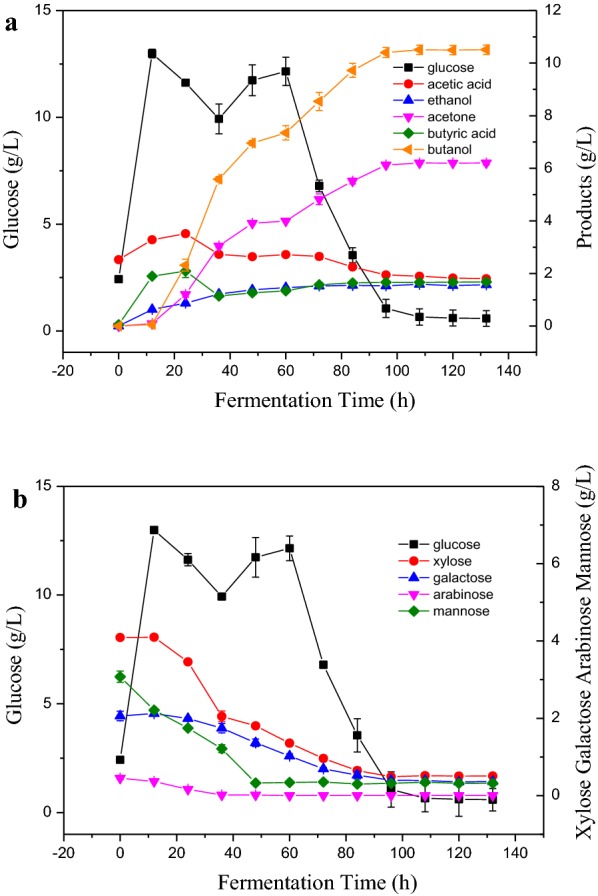
Effect of adding detoxified prehydrolysates on ABE production with OPLP-W in SSF process

Considering the removal of residual extractable lignin in SSF gave the best ABE production (Table 1), detoxified prehydrolysates was supplemented into OPLP-W in SSF processes. Two-step detoxification has been used to detoxify the prehydrolysates from organosolv pretreatment [43]. The final butanol and ABE concentration in OPLP-W with prehydrolysate (OPLP-W/PH) reached 10.51 g/L and 18.29 g/L, respectively. No “acid crash” occurred in this case (Fig. 4a). It suggested that adding detoxified prehydrolysates into SSF processes alleviated the “acid crash” for ABE fermentation due to the increase of initial sugar concentration. The acetone and ethanol reached 6.13 and 1.53 g/L at 96 h, respectively. The residual glucose was only 0.59 g/L at 132 h. The organism began to produce butyric and acetic acids at 12 h. Butyric acid peaked (2.09 g/L) at 24 h and then decreased to 1.14 g/L at 36 h and gradually increased to 1.68 g/L at 132 h. The butyric acid maximum concentration was 35% less than that from OPLP-W without prehydrolysates in the previous SSF process. Butanol production from OPLP-W/PH began at 24 h and slowed at 48 h, but it recommenced at 60 h quickly. The ABE fermentation from OPLP-W/PH completed within 96 h, which was 60 h shorter than that from OPLP-W without prehydrolysates in the SSF process (Fig. 3b). These results indicated the addition of sugars from prehydrolysates potentially prevented the “acid crash” and helped the ABE fermentation in the SSF processes.

After adding prehydrolysates into OPLP-W, the initial glucose, xylose, mannose, galactose concentration in the aqueous phase was 2.06, 4.09, 3.08 and 2.42 g/L, respectively (Fig. 4b). The released glucose reached 12.99 g/L at 12 h and then decreased to 9.93 g/L at 36 h. After that, the glucose concentration increased to 12.15 g/L at 60 h, then decreased again quickly to 1.06 g/L at 96 h. The glucose concentration at 12 h was 13% lower than that without prehydrolysates in the previous SSF (Fig. 3b). This probably was caused by the inhibition resulting from residual undetoxified inhibitors in prehydrolysates. It is reported that lignin-derived aromatic compounds induced inhibition or complete inactivation of enzymes [20]. The initial sugars in the prehydrolysates could also inhibit the enzymatic hydrolysis. Previously, sugar inhibition on cellulases and beta-glucosidase has been reported on enzymatic hydrolysis of softwood substrates [44]. The solventogenic clostridia are capable of using both hexose and pentose as carbon source for ABE production. It was observed that mannose was used firstly and quickly, followed by xylose. All the available sugars were assimilated by *Clostridium* at the end of fermentation, leaving an insignificant amount of residual sugars. The results suggested the prehydrolysates could reduce the ABE fermentation time in SSF processes, all the C5 and C6 could be consumed for ABE production and the “acid crash” could be potentially avoided.

In this study, the ABE production from OPLP-W and OPLP-UW was carried out in two different fermentation processes. Overall, the SSF process gave a higher ABE production (15.74 g/L) compared to the SHF process (11.89 g/L) due to the removal of glucose inhibition in SSF process. Although fermentation time for the SSF (156 h) was longer than the SHF (96 h), the whole time of SHF was identical to SSF if the time for enzymatic hydrolysis (72 h) was taken into consideration. The residual extractable lignin showed a significant effect on ABE fermentation on OPLP-W and OPLP-UW. In SHF process, it prevented the “acid crash” by slowing the microbial metabolism and increased the ABE yield from 0.04 g/g (OPLP-W) to 0.20 g/g (OPLP-UW). However, in the SSF process, whereas the initial sugar concentration was low, the presence of residual extractable lignin intensified the “acid crash” and the ethanol-washed substrate (OPLP-W) resulted in higher ABE production than from OPLP-UW (15.74 g/L vs. 3.65 g/L). Also, the addition of prehydrolysates to OPLP-W further improved the ABE fermentation by prevention of “acid crash” and gave the highest ABE titer of 18.29 g/L.

## Conclusions

The effect of residual extractable lignin in ABE fermentation of organosolv**-**pretreated loblolly pine has been compared in SHF and SSF processes. Unexpectedly, the extractable lignin in OPLP-UW and OPLP-W/EOL was observed to slow down the metabolic activity of clostridium and prevent the “acid crash” in SHF processes. The presence of residual extractable lignin enhanced the final butanol concentration compared to the OPLP-W. However, the extractable lignin did not help the ABE fermentation in SSF process and intensified the “acid crash”. This is caused by the inhibition of lignin to both saccharification and fermentation. The removal of residual extractable lignin by ethanol washing is needed to reduce its inhibitory effect on ABE fermentation in SSF. The low initial sugar concentration in SSF process could be a possible reason for acid crash. The addition of prehydrolysates could potentially prevent the “acid crash” of ABE fermentation in SSF processes by increasing the initial sugar concentration. It also significantly shortened the fermentation time from 156 h to 96 h and improved the efficiency of lignocellulose by using the sugar dissolved in the aqueous phase.

## Materials and methods

### Chemicals and microorganisms

Glucose and NaOH were purchased from VWR (West Chester, PA). Ca(OH)_2_, *p*-aminobenzoic acid and CH_3_COONH_4_ were purchased from Alfa Aesar (Heysham, England). Thiamine was purchased from Alfa Aesar (Ward Hill, MA). Dowex 1X4 resin (chloride form) and biotin were purchased from Sigma-Aldrich (St. Louis, MO). H_2_SO_4_ (98%) and NaCl were purchased from VWR (West Radnor, PA). K_2_HPO_4_, KH_2_PO_4_, MgSO_4_·7H_2_O, MnSO_4_·H_2_O, FeSO_4_·7H_2_O were purchased from Fisher Scientific (Fair Lawn, NJ). Citric acid was purchased from Mallinckrodt Chemicals (Phillipsburg, NJ). CaCO_3_ was purchased from EMD Chemicals (Gibbstown, NJ). Reinforced Clostridial Broth medium (RCM) was purchased from HIMEDIA laboratories (Mumbai, India). Cellic CTec 2 was obtained from Novozymes North America, Inc (Franklinton, UC). DI water was produced by the Barnstead Nanopure UV Ultrapure Water System (Thermo Fisher Scientific, Marietta, OH).

*Clostridium acetobutylicum* ATCC 824 was used for butanol production. It was routinely stored as spores at 4 °C and treated by heat shock at 75 °C for 10 min followed by cooling down in an ice bath prior to cultivation. The RCM medium was sparged with nitrogen and then autoclaved at 121 °C for 15 min. The heat-shocked cells were grown until the optical density (OD) reached 1.30 determined by an UV–vis spectrometer (Thermo Scientific, Madison, WI) at 600 nm.

### Organosolv pretreatment

Loblolly pine wood chips were collected by the Forest Products Laboratory at Auburn University and those free of barks and size of 1.0 × 1.0 cm (L × W) were selected for organosolv pretreatment. Wood chips (80 g, oven-dry weight) were soaked in 65% (*v/v*) ethanol solution with 1.1% (*w/w*) sulfuric acid (on the basis of biomass dry weight) overnight (7:1 liquor/solid ratio) and then loaded into a 1-L Parr reactor (Parr Instrument Co., Moline, IL) to be treated at 170 °C for 60 min. The spent liquor (aqueous phase) was separated from solid by vacuum filtration upon the completion of pretreatment. Afterward, if ethanol washing was needed, the solid fraction was washed by 700 mL warm ethanol solution (65% (*v/v*), 50 °C) three times to dissolve the ethanol extractable lignin and followed by washing by 700 mL DI water four times to remove the residual ethanol. The cellulose-rich solid fraction was homogenized in a blender for 15 s and then used for fermentation and the aqueous phase was subject to detoxification. They were both stored at 4 °C until use.

The EOL was collected from the spent liquor and the ethanol washes. Threefold DI water was added to precipitate lignin and then the lignin fraction was separated by filtration and then washed thoroughly with DI water, dried in air and then in the oven (105 °C). Sample from the mixture of filtrate and water washes was taken to determine the water-solubles. The collected materials include 39.0 g wood pulp, 14.2 g EOL and 14.2 g water-solubles after pretreatment of 80 g oven-dry wood. The water-solubles contained 9.50 g carbohydrates, 2.31 g acid-soluble lignin, 0.35 g HMF, 0.71 g furfural, and 1.37 g acetic acid.

### Chemical analysis of raw biomass and pretreated OPLP

The extractives content in raw biomass, organosolv-pretreated OPLP-UW and OPLP-W (unwashed OPLP and washed OPLP) was determined as previously described [45]. The composition analysis of carbohydrate and lignin before and after ethanol organosolv pretreatment was carried out using extractives-free samples as previously described [46]. The sugar content of prehydrolysate was determined according to NREL standard method, NREL/TP-510-42623 [47]. The chemical composition of untreated and ethanol organosolv-pretreated loblolly pine is shown in Table 2.

**Table 2:** Chemical composition of untreated and ethanol organosolv-pretreated loblolly pine

	Untreated (%)	Organosolv treated	
		OPLP-UW (%)	OPLP-W (%)
Glucan	42.30 ± 0.38	72.74 ± 0.20	82.14 ± 0.03
Xylan	7.51 ± 0.05	2.17 ± 0.01	1.69 ± 0.08
Galactan	2.96 ± 0.05	0.36 ± 0.03	0.40 ± 0.02
Arabinan	1.78 ± 0.03	0.63 ± .02	0.69 ± 0.05
Mannan	11.17 ± 0.08	1.36 ± 0.00	0.99 ± 0.02
Ethanol extractives	1.18 ± 0.05	9.64 ± 0.12	0.79 ± 0.04
Acid insoluble lignin (AIL)	29.45 ± 0.27	12.11 ± 0.15	13.52 ± 0.10
Acid-soluble lignin (ASL)	0.56 ± 0.05	0.28 ± 0.00	0.35 ± 0.01
Ash	0.36 ± 0.02	0.03 ± 0.00	0.04 ± 0.00
Total	97.27	99.31	100.61

### Enzymatic hydrolysis

Cellic CTec 2 was used in enzymatic hydrolysis of pretreated biomass and its filter paper enzyme activity was 126 FPU/mL. Enzymatic hydrolysis of OPLP-W and OPLP-UW (moisture content, ~ 70%) was carried out in 125-mL serum bottle with a working volume of 50 mL with glucan loading of 5.8% (*w/v*). Two different conditions were performed with both OPLP-W and OPLP-UW for SHF and SSF conditions, respectively: (1) pH 4.8 controlled by 50 mM citrate buffer, 50 °C and 150 rpm; (2) pH 6.0 controlled by adding 0.25 g CaCO_3_, 35 °C and 80 rpm. To study the effect of lignin on enzymatic hydrolysis, 0.3 g EOL (equivalent to the amount of lignin removed by washing) was added into the ethanol-washed substrate prior to enzymatic hydrolysis. The mixture was autoclaved at 121 °C for 15 min, and then after cooling to room temperature, the enzyme was added to initiate the hydrolysis. Enzyme loading was 25 FPU/g glucan. Samples were taken aseptically to prevent contamination. The enzymatic hydrolysis yield was calculated as glucose released during hydrolysis divided by theoretical total glucose in the substrate.

### Separate hydrolysis and fermentation (SHF)

For the SHF process, the mixture obtained from enzymatic hydrolysis (pH 4.8 controlled by 50 mM citrate buffer, 50 °C and 150 rpm) was applied for fermentation. The volume of the mixture after enzymatic hydrolysis became 45 ml due to the loss in the sample taken. It was brought to 50 mL after inoculation (10% *v/v*) and glucan loading became 5.2% (*w/v*). Upon completion of enzymatic hydrolysis, it was supplemented with previous filter-sterilized nutrients stock: 50 μL vitamins (*p*-aminobenzoic acid, 1 g/L, thiamine, 1 g/L, biotin, 0.01 g/L), 0.25 mL minerals (MgSO_4_·7H_2_O, 40 g/L, MnSO_4_·H_2_O, 2 g/L, FeSO_4_·7H_2_O, 2 g/L, NaCl, 2 g/L) and 0.5 mL buffer (K_2_HPO_4_, 50 g/L, KH_2_PO_4_, 50 g/L, CH_3_COONH_4_, 220 g/L). CaCO_3_ (0.25 g) was added into the broth to control the pH during fermentation. Then the mixture in serum bottle was vacuumed and flushed with nitrogen for 7 cycles to remove oxygen by using a purge valve. The fermentation was initiated by adding 5 mL inoculum (10% inoculation).

### Simultaneous saccharification and fermentation (SSF)

The SSF process of both OPLP-W and OPLP-UW was carried out in 125-mL serum bottle with working volume of 50 mL with glucan loading of 5.2% (*w/v*). They were autoclaved at 121 °C for 15 min and then supplemented with previous filter-sterilized nutrients stock the same as SHF process listed above. CaCO_3_ (0.25 g) was added into the broth. Then the slurry in the serum bottle was vacuumed and flushed with nitrogen for 7 cycles to remove oxygen by using a purge valve. The enzyme loading was 25 FPU/g glucan and the enzyme was sterilized by passing through a 0.2-µm membrane filter. The fermentation was initiated by adding enzyme and 5 mL inoculum (10% inoculation). Both SHF and SSF were carried out at 35 °C and 80 rpm.

### SSF process supplemented with detoxified prehydrolysates

Ethanol in prehydrolysate was evaporated at 40 °C in a rotary evaporator (IKA RV10 basic) and the pH was adjusted to 4.0 with NaOH before evaporation. The concentrated prehydrolysate was then diluted with DI water to make the total volume the same as that before evaporation. Two-step detoxification was carried out as described previously [43]. Briefly, the pH of prehydrolysate was adjusted to 10 by adding Ca(OH)_2_ and incubated at 90 °C and 100 rpm for 30 min. Afterward, 10 g activated Dowex 1X4 resin was added to 100 mL prehydrolysate and the whole mixture was incubated at 25 °C and 100 rpm for 1 h. The liquid was separated from the resin by centrifuge at 4000 rpm for 10 min and then the pH was adjusted back to 7 with H_2_SO_4_. The detoxified prehydrolysate was supplemented into SSF in place of water.

All fermentations were performed in duplicates. Samples were taken periodically for sugar and ABE analysis. Butanol yield was calculated as butanol produced divided by glucose content in the pretreated substrate (and prehydrolysate if applicable) and is expressed as g/g. ABE yield was calculated as the total ABE produced divided by glucose content in the pretreated substrate (and prehydrolysate if applicable) and is expressed as g/g.

### Sugars and products analysis

The sugar content was quantified by a Shimadzu (LC-20A) HPLC system consisting of a degasser, autosampler, LC-20AD pump, and RID-10A detector, equipped with a 300 mm × 7.8 mm i.d., 9 µm, Aminex HPX-87P column and a 30 mm × 4.6 mm i.d. guard column of the same material (Bio-Rad, Hercules, CA). Nano-pure water was used as a mobile phase running at 0.6 mL/min. The column temperature was maintained at 85 °C. Acetic acid, butyric acid, ethanol, acetone, butanol, HMF, and furfural were quantified by the same HPLC system (Shimadzu LC-20A) equipped with an Aminex HPX-87H column. The mobile phase was composed of 5 mM of sulfuric acid running isocratic at 0.6 mL/min. The column temperature was kept at 45 °C.

## Data Availability

All data generated or analyzed during this study are included in this published article.

## Abbreviations

ABE: Acetone–butanol–ethanol
SHF: Separate hydrolysis and fermentation
SSF: Simultaneous saccharification and fermentation
OPLP: Organosolv-pretreated loblolly pine
OPLP-UW: Organosolv-pretreated loblolly pine—unwashed
OPLP-W: Organosolv-pretreated loblolly pine—washed
OPLP-W/PH: Washed OPLP with detoxified prehydrolysates
EOL: Ethanol organosolv lignin
NREL: National Renewable Energy Lab
